# Growth Inhibitory Efficacy of Chinese Herbs in a Cellular Model for Triple-Negative Breast Cancer

**DOI:** 10.3390/ph14121318

**Published:** 2021-12-17

**Authors:** Nitin T. Telang, Hareesh B. Nair, George Y. C. Wong

**Affiliations:** 1 Cancer Prevention Research Program, Palindrome Liaisons Consultants, Montvale, NJ 07645, USA; 2 Department of Obstetrics and Gynecology, University of Texas Health Sciences Center, San Antonio, TX 78229, USA; nairh@uthscsa.edu; 3 Breast Center, Maimonides Medical Center, Brooklyn, NY 11219, USA; drgeorgewong@yahoo.com; 4 American Foundation for Chinese Medicine, New York, NY 11103, USA

**Keywords:** breast cancer, growth inhibition, Chinese herbs

## Abstract

Triple-negative breast cancer (TNBC) is characterized by the absence of estrogen receptor-α progesterone receptor and human epidermal growth factor receptor-2. Treatment for this breast cancer subtype is restricted to multidrug chemotherapy and survival pathway-based molecularly targeted therapy. The long-term treatment options are associated with systemic toxicity, spontaneous and/or acquired tumor resistance and the emergence a of drug-resistant stem cell population. These limitations lead to advanced stage metastatic cancer. Current emphasis is on research directions that identify efficacious, naturally occurring agents representing an unmet need for testable therapeutic alternatives for therapy resistant breast cancer. Chinese herbs are widely used in traditional Chinese medicine in women for estrogen related health issues and also for integrative support for cancer treatment. This review discusses published evidence on a TNBC model for growth inhibitory effects of several mechanistically distinct nontoxic Chinese herbs, most of them nutritional in nature, and identifies susceptible pathways and potential molecular targets for their efficacy. Documented anti-proliferative and pro-apoptotic effects of these herbs are associated with downregulation of RB, RAS, PI3K, and AKT signaling, modulation of Bcl-2/BAX protein expressions and increased caspase activity. This review provides a proof of concept for Chinese herbs as testable alternatives for prevention/therapy of TNBC.

## 1. Introduction

Progression of breast cancer to advanced stage metastatic disease represents a major cause of mortality in women. The American Cancer Society projects the incidence of newly diagnosed female breast cancer as 281,550 and cancer related deaths as 43,600 in 2022 [1]. Amongst the molecular subtypes of breast cancer, the triple-negative breast cancer (TNBC) represents an aggressive subtype that is diagnosed in about 20% of the new breast cancer cases [2]. TNBC lacks the expressions of estrogen receptor-α (ER-α), progesterone receptor (PR) and of amplified human epidermal growth factor recptor-2 (HER-2), and is at high risk for developing chemo-resistance and distant metastasis [3]. At the molecular levels TNBC exhibits aberrant signaling of the tumor suppressor RB gene, and activation of rat sarcoma (RAS), phosphoinositide 3 kinase (PI3K) and protein kinase B (AKT) signaling pathways that facilitate hyper-proliferation, increased migration/invasion and subsequent survival of cancer cells [4].

Conventional chemotherapy includes treatment with cytotoxic agents such as anthracyclins, cisplatins and taxols, endocrine therapy includes treatment with selective estrogen receptor modulators such as tamoxifen and raloxifene, treatment with selective estrogen receptor disruptors such as fulvestrant, treatment with aromatase inhibitors such as letrozol and exemestane, and HER-2 targeted therapy includes treatment with small molecule inhibitors such as lapatinib and neratinib. These options represent treatments of choice for the hormone responsive Luminal A and Luminal B subtypes. In contrast, because of lack of expressions of hormone and growth factor receptors, TNBC does not respond to the aforementioned options. The treatment of choice for this subtype includes multi-drug chemotherapy containing anthracyclins, taxols and cisplatin, or small molecule inhibitor-based RAS, PI3K, AKT and mammalian target of rapamycin (mTOR) pathway selective targeted therapy [5].

The long-term treatment options for molecular subtypes of breast cancer including TNBC are frequently associated with systemic toxicity, spontaneous or acquired tumor resistance and emergence of a therapy resistant cancer stem cell population, all of which favor progression of therapy resistant disease [6]. These limitations of long-term therapeutic options emphasize investigations that focus on identification of efficacious non-toxic agents that display distinct specificity and selectivity for cancer cells. Such promising agents may represent testable alternatives for therapy resistant breast cancer.

Several pharmacological agents that function via distinct mechanism of action have documented preventive efficacy in preclinical models of breast cancer [7]. These agents at their respective therapeutic doses exhibit dose limiting systemic toxicity and adverse side effects. In contrast, dietary phytochemicals present in vegetables and fruits are unlikely to possess adverse systemic toxicity [8], and therefore, may represent testable alternatives against chemo-endocrine therapy. Growth inhibitory effects of naturally-occurring bioactive agents such as polyphenols, flavones, phytoalexins, terpenes, coumarins and saponins, as well as those of vitamins and micro-nutrients have been documented [9,10].

A number of herbs commonly used in traditional Chinese medicine have exhibited anti-proliferative and pro-apoptotic effects in a cellular model for the Luminal A breast cancer subtype [11,12,13] and also in a model for the triple-negative breast cancer subtype [14,15,16]. At the mechanistic levels, the anti-proliferative effects of the herbs are associated with inhibition of cell cycle progression, and modulated expression of cell cycle regulatory proteins functioning at G_1_ and/or G_2_ phases of the cell cycle. The pro-apoptotic effects of the herbs are associated with upregulation of apoptotic cell population at the sub G_0_ phase of the cell cycle, modulation of the expressions of apoptotic proteins Bcl-2 and BAX and induction of pro-apoptotic caspase activity, the latter representing a specific marker of the intrinsic apoptotic process.

Chinese herbs, many of them nutritional in nature, have been traditionally used for the management of general health concerns and also for women’s estrogen health issues, including breast diseases. These herbs lack clinical systemic toxicity, and because of their nontoxic nature and documented human use [17,18,19,20], may represent testable alternatives for secondary prevention/therapy of TNBC.

The present review provides an overview of conventional and targeted chemo-endocrine therapy and their limitations, and effective dietary phytochemicals and Chinese nutritional herbs and their advantages over chemotherapy. In addition, this review discusses the evidence for validation of an experimental approach using a cellular model for TNBC to evaluate the growth inhibitory efficacy of Chinese herbs. The experiments are focused on (i) the development and characterization of a cellular model for TNBC, (ii) the screening of Chinese herbs for their growth inhibitory efficacy and (iii) identifying potential mechanistic leads. These research directions have provided mechanistic leads to suggest that Chinese herbs may represent nontoxic testable alternatives for secondary prevention/therapy of TNBC.

## 2. Experimental Model

The human breast carcinoma derived MDA-MB-231 cells lack the expressions of ERα, PR and amplified HER-2 [21,22]. This cell line represents a preclinical cellular model for the TNBC subtype of clinical breast cancer.

To examine specific growth characteristics of the TNBC model, comparative experiments were performed to monitor the status of proliferative end points in the tumorigenic triple- negative MDA-MB-231 cells and the non-tumorigenic triple negative 184-B5 cells and the data as summarized from [14,15,16,17,18,19,20,21,22,23] demonstrate that relative to the non-tumorigenic cells, the tumorigenic cells exhibit a decrease in the population doubling time, and in the ratio between quiescent and proliferative cells, while saturation density exhibits an increase in favor of the tumorigenic cells. Anchorage independent (AI) growth represents a specific and sensitive in vitro marker for in vivo tumor growth. The data on AI colony number as summarized from [14,15,16,17,18,19,20,21,22,23], also exhibit a substantial increase in the tumorigenic cells (Table 1).

These data provide evidence that the hyper-proliferative tumorigenic cells maintain a persistent risk of developing cancer.

### 2.1. Mechanistic Assays

The mechanistic assays have utilized optimized and published protocols for cell viability measuring viable cell number, AI colony formation measuring AI colony number, cell cycle progression measuring the cell population in G_1_, S and G_2_/M phases of the cell cycle, cellular apoptosis measuring the cell population in the sub G_0_ phase of the cell cycle, and pro-apoptotic caspase 3/7 activity measuring relative luminescent units (RLU). In addition, the Western blot based protein expression assay was used to monitor the activation of RB, RAS, PI3K and AKT pathways as determined from the ratio of phosphorylated: total proteins.

### 2.2. Test Agents

A selection of nontoxic Chinese herbs have demonstrated efficacy against hormone receptor positive, HER-2 receptor negative human mammary carcinoma derived MCF-7 cells [12]. These herbs are used as test agents in the present model for TNBC. The origin of the source for the test agent and major constituent bio-active agents that have published growth inhibitory evidence are presented in Table 2.

It is conceivable that the content of bioactive agents in the herbs is likely to be highly variable because of differing climatic and soil conditions, and anti-proliferative effects of individual agents on cancer cells may involve distinct mechanisms of action [11,12,13,14,15,16].

The herbal formulations for the clinical use that contain several herbs in combination are routinely brewed in water to prepare herbal tea for patient consumption. Therefore to simulate clinical condition, non-fractionated aqueous extracts of the herbs, as presented in Table 2, are used for the present experiments. The aqueous extracts are prepared following an optimized water extraction method that is used routinely in the published studies [11,12,13,14,15,16]. This procedure is consistent with the method of preparation for patient consumption. The stock solutions of the aqueous extracts were diluted in the culture medium at the concentrations of 1 mg/mL. These stock solutions were used for treatment at µg/mL concentrations.

A comparative study using an isogenic model with modulated function of estrogen receptor-α (ER-α) function demonstrated that based on their preferential growth inhibitory effects, Chinese herbs may be selectively efficacious on ER functional (ER-F) or ER non-functional (ER-NF) phenotype [12]. Promising herbs effective for the ER-NF phenotype provided a rationale to examine their growth inhibitory efficacy in the present TNBC model.

### 2.3. Anti-Proliferative Effects of Chinese Herbs

In the experiments to examine growth inhibition in response to treatment with the herbs, a viable cell number was determined using the growth inhibitory dose response assay of seven day duration. These dose response experiments provided a basis to determine the half-maximum (IC_50_) and the maximum cytostatic (IC_90_) concentrations.

The data presented in Table 3 indicate that CO, PC, DA, LB, VY and EU are the most effective herbs for inhibiting the growth, exhibiting an effective IC_50_ concentration range of 1 µg/mL to 21 µg/mL, respectively. LL, CS and EG exhibited a concentration range of 88 µg/mL to 102 µg/mL. In contrast, DF and TM exhibited a substantially higher IC_50_ concentrations of 650 µg/mL and 800 µg/mL, respectively, While TM exhibited only a limited efficacy (IC_50_ > 1000 µg/mL) against TNBC cells.

The data presented in Table 4 indicate that CO, PC, DA, LB and EU are the most effective herbs, exhibiting effective IC_90_ concentrations ranging from 5 µg/mL to 38 µg/mL, respectively. VY and LL exhibited IC_90_ concentrations of 200 µg/mL and 274 µg/mL. CS and DF exhibited a substantially higher IC_90_ concentrations of 671 µg/mL and 1000 µg/mL, while EG, SG and TM exhibited only a limited efficacy (IC_90_ > 1000 µg/mL). Percent proliferating tumor cell population represent the cells that survive in response to the treatment with the herbs. This cell population ranges from 9.7% to 14.7% of the untreated control cells.

The data presented in Table 4 indicate that CO, PC, DA, LB and EU are the most effective herbs, exhibiting effective IC_90_ concentrations ranging from 5 µg/mL to 38 µg/mL, respectively. VY and LL exhibited IC_90_ concentrations of 200 µg/mL and 274 µg/mL. CS and DF exhibited a substantially higher IC_90_ concentrations of 671 µg/mL and 1000 µg/mL, while EG, SG and TM exhibited only a limited efficacy (IC_90_ > 1000 µg/mL).

AI colony formation: The long-term growth inhibitory effects of the herbs at their respective IC_90_ concentrations were examined using the AI colony formation assay. The number of AI colonies formed at day 21 after seeding provided the quantitative end point. The data were expressed as mean AI colony number ± SD. It is notable that DA, PC and CO at their respective IC_90_ concentrations exhibited a range of about 93% to 84% inhibition, respectively. In contrast, LL, DF, SG, LB, VY, EU and TM exhibited a range of about 55% to 47% inhibition respectively, and CS and EG were not effective (Table 5).

The AI colony formation represents an in vitro end point for growth that is specific for tumorigenic cells, and exhibits a positive correlation with the tumor formation in vivo [25]. Thus, the AI growth assay has been utilized as an in vitro surrogate end point biomarker for cancer risk [11,12,13,14,15,16]. The data on the reduction in AI colony number therefore suggest that the Chinese herbs may effectively reduce breast cancer risk.

In herbal formulations for TNBC patients, 15 g of CO, PC, DA, LB, EU and VY in dry raw herb form are usually included as component herbs. For LL, CS, EG and DF, at least 30 g of each are necessary to achieve a comparable outcome. At these concentrations the herbs do not exhibit toxic side effects (Dr. George YC Wong, Personal communication). This clinical observation is consistent with the growth inhibitory effects exhibited by the herbs tested at their respective IC_50_ and IC_90_ concentrations in the cell viability assay. The IC_90_ (maximally cytostatic) concentrations are defined as the highest concentrations that result in the number of viable cells that are higher than the initial seeding density. The toxic concentrations are defined as the concentrations that result in the number of viable cells that are lower than the initial seeding density. The herbs at their respective toxic concentrations have not been used. The present preclinical data and the clinical observations, taken together, indicate that these Chinese herbs are within the effective nontoxic range for TNBC.

The growth inhibitory effects of the Chinese herbs have provided a rationale for the experiments focused on cell cycle progression and cellular apoptosis that may provide leads for the mechanisms of action and identification of potential molecular targets for their efficacy.

**Figure 1:**
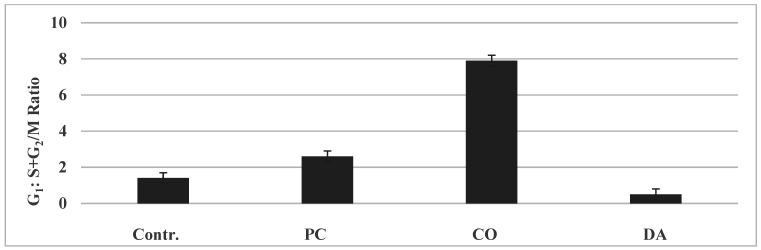
Effect of Chinese herbs on cell cycle progression. Treatment with PC and CO at their respective IC_90_ doses increased the ratio, while treatment with DA at IC_90_ dose reduced the ratio. The data are presented as G_1_: S + G_2_/M ratio mean ± SD, *n* = 3 per treatment group, and analyzed by ANOVA with Dunnett’s multiple comparison test (α = 0.05). Contr., control; PC, *Psoralea corylifolia*; CO, *Cornus officinalis*. SD, standard deviation; ANOVA, analysis of variance. Data summarized from [14,15,16].

Cell cycle progression: The experiment presented in Figure 1 demonstrated that PC and CO increased the G_1_: S + G_2_/M ratio, while DA induced a reduction in this ratio. These changes are due to selective G_1_ or G_2_ arrest, respectively. Data summarized from [14,15,16].

The data as summarized from [14,15,16] suggest that the herbs affect distinct phases of the cell cycle important for either G_1_ to S phase transition or G_2_ to M phase transition of the cycling cells. These data provide a rationale to examine the status of G_1_ specific and G_2_ specific signaling molecules as mechanistic leads.

RB signaling: The tumor suppressor RB gene plays an important role in the regulation cell cycle progression by affecting the G_1_ to S phase transition and functions via the cyclin D-CDK4/6-pRB/E2F signaling cascade. The post-translational modification of RB by phosphorylation, hyper-phosphorylation-induced release of E2F transcription factor and expression of RB target genes are established mechanisms of action [26,27]. The TNBC molecular subtype is notable for defective RB function where pRB status represents a marker for abnormal tumor suppressive function of the RB gene [28,29]. During RB signaling the status of phosphorylation is altered, while that of the total protein remains essentially unchanged. Thus, phosphorylated: total protein ratio represents an important marker for pathway activation [26,29].

**Figure 2:**
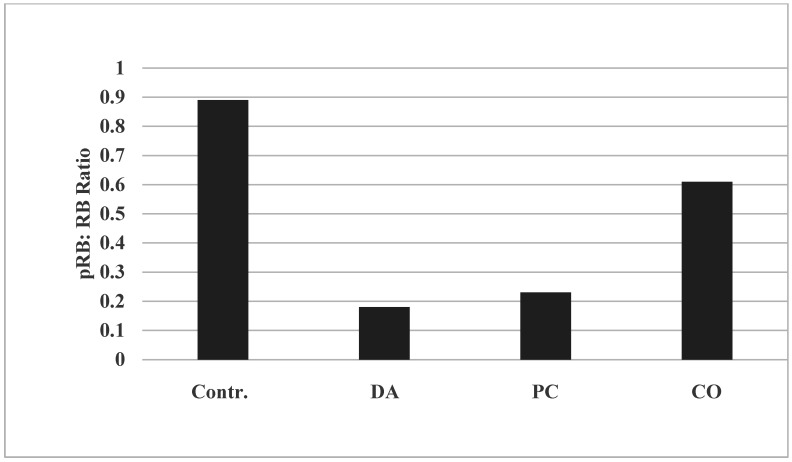
Effect of Chinese herbs on RB Signaling. Treatment with DA, PC and CO at their respective IC_90_ doses decreased the pRB: RB ratio. Data presented as arithmetic means of ASU from duplicate determinations. pRB, phosphorylated RB; RB, retinoblastoma; ASU, arbitrary scanning unit; DA, *Dipsacus asperoides*; PC, *Psoralea corylifolia*; CO, *Cornus officinalis*. Data summarized from [14,15,16].

The data demonstrate that treatment with DA resulted in about an 80% inhibition in pRB. Treatment with PC resulted in about a 74% inhibition in pRB. Treatment with CO resulted in about a 31% inhibition in pRB, relative to the untreated control (Figure 2). Thus, reduction of pRB: RB ratio may be indicative of the efficacy of these herbs to inhibit aberrant RB signaling. Based on the inhibition in the pRB; RB ratio, these data provided a rank order of DA > PC > CO. Data summarized from [14,15,16].

Cyclin dependent kinase inhibitors: Cyclin dependent kinases CDK4 and CDK6 are critical for G_1_ to S phase transition [30]. Small molecule inhibitors selective for CD4/CDK6, such as palbociclib and ribociclib, have shown clinical efficacy in the ER positive/HER-2 negative metastatic breast cancer, and are used in combination with small molecule inhibitors of aromatase activity [31,32,33]. Notably, efficacy of the CDK4/6 inhibitors has also been demonstrated in TNBC [34,35,36,37]. However, clinical use of CDK4/6 inhibitors is associated with systemic toxicity and acquired tumor resistance.

**Figure 3:**
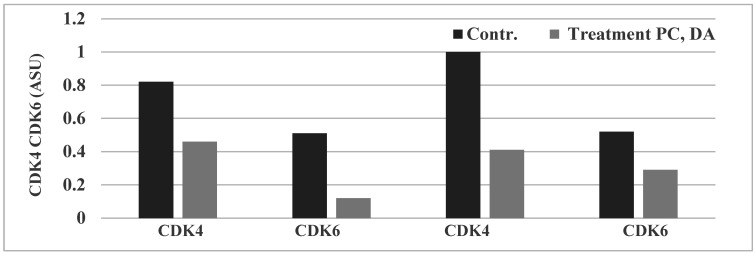
Effect of Chinese herbs on CDK4 and CDK 6 expression. Treatment with PC and DA at their respective IC_90_ doses inhibited the expressions of CDK4 and CDK6. Data presented as arithmetic means of ASU from duplicate determinations. CDK, cyclin dependent kinase; ASU, arbitrary scanning unit; Contr., Control; Treatment: PC, *Psoralea corylifolia*; DA, *Dipsacus asperoides*. Data summarized from [15,16].

The data demonstrate that treatment with PC results in in about a 38% inhibition of CDK4 and about a 74% inhibition of CDK6. Treatment with DA results in about a 48% inhibition of CDK4 and about a 29% inhibition of CDK6 (Figure 3). Data summarized from [15,16].

Collectively, these data suggest that PC and DA may function as natural inhibitors of CDK4 and CDK 6, and thereby, may provide testable alternatives against the clinical limitations of toxicity and tumor resistance.

RAS, PI3K, AKT signaling: Activation of the oncogenic RAS gene represents a common event in signaling via the RAF-MEK-ERK (MAPK pathway) or via the PI3K-AKT pathway [38]. These two pathways represent the survival pathways commonly activated in cancer cells. The phosphorylated protein: total protein ratio represents an important marker of activation of these signaling pathways. Activation of MEK, ERK PI3K and AKT represent therapeutic targets that respond to clinically efficacious small molecule inhibitors [39,40,41,42].

**Figure 4:**
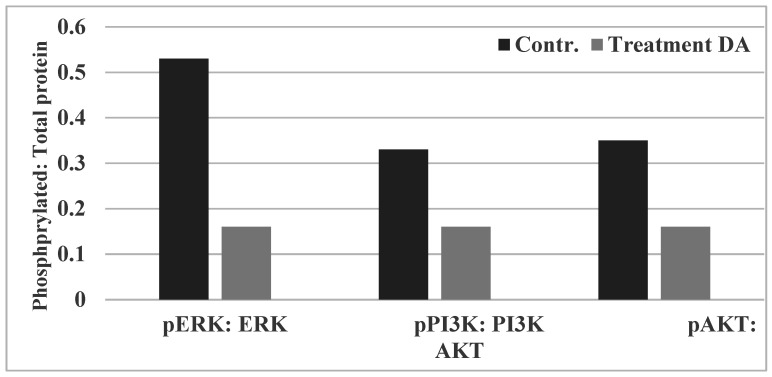
Effect of Chinese herbs on RAS, PI3K, and AKT signaling. Treatment with DA at its IC_90_ dose inhibited the phosphorylated: total protein ratio. Data presented as arithmetic means of phosphorylated: total protein ratio of ASU from duplicate determinations. ERK, extracellular receptor kinase; PI3K, phosphatidylinositol 3-kinase, AKT, Protein kinase B; ASU, arbitrary scanning unit. Contr, control; Treatment: DA, *Dipsacus asperoides*. Data summarized from [16].

These data on the status of ERK, PI3K and AKT signaling demonstrate that treatment with DA results in about a 70% reduction in the pERK: ERK ratio, about a 51% reduction in the pPI3K: PI3K ratio and about a 54% reduction in the pAKT: AKT ratio, relative to the control (Figure 4). Data summarized from [16].

Collectively, these data suggest that DA inhibits RAS, PI3K and AKT signaling. In this context it is notable that rosemary extract inhibits AKT and mTOR signaling in the TNBC cells [43], and lycopene inhibits the PI3K/AKT/mTOR pathway in oral cancer cells via reducing the phosphorylation of PI3K, AKT and mTOR [44].

Cellular apoptosis: During normal homeostatic growth control in non-tumorigenic cells, proliferation and apoptosis are stringently regulated [45]. In hyper-proliferative tumorigenic cells, the apoptotic pathways are frequently downregulated, and induction of cellular apoptosis by chemotherapeutic agents represents a marker for their efficacy [46,47,48].

The pro-apoptotic effects of the herbs were examined by determining the status of the sub G_0_ (apoptotic) cell population and by the extent of induction of pro-apoptotic caspase 3/7 activity.

**Figure 5:**
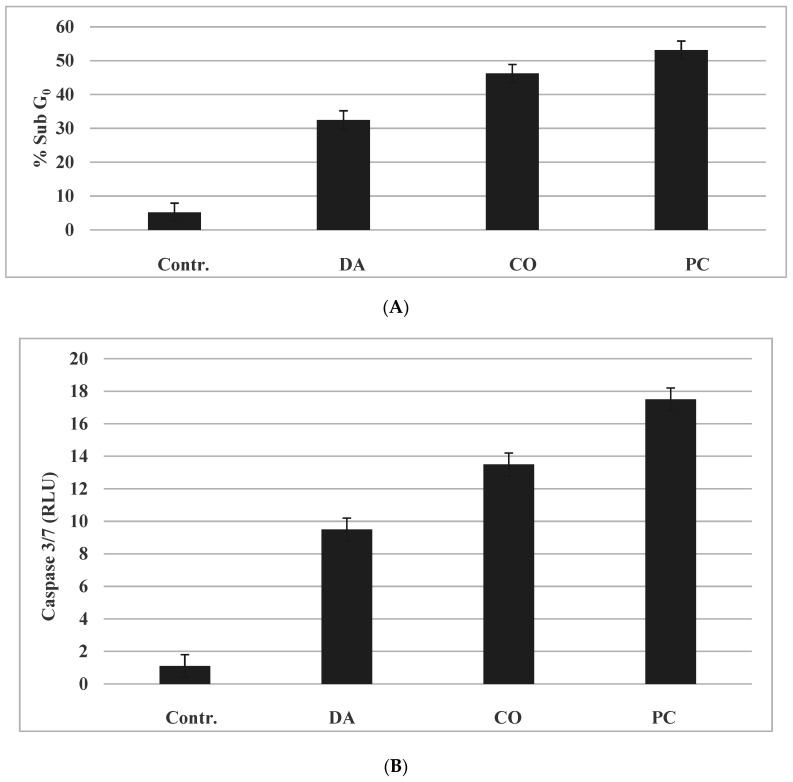
(**A**): Effect of Chinese herbs on cellular apoptosis. Treatment with DA, CO and PC at their respective IC_90_ doses increased % sub G_0_ population. The data are presented as % sub G_0_ population mean ± SD, *n* = 3 per treatment group. (**B**): Effect of Chinese herbs on Caspase 3/7 activity. Treatment with DA, CO and PC at their respective IC_90_ doses increased caspase 3/7 activity. The data are presented as RLU mean ± SD, *n* = 3 per treatment group. The data are analyzed by ANOVA with Dunnett’s multiple comparison test. (α = 0.05). Contr.; control; DA, Dipsacus asperoides; CO, Cornus officinalis; PC, Psoralea corylifolia; RLU, relative luminescent unit; SD, standard deviation; ANOVA, analysis of variance. Data summarized from [14,15,16].

The data demonstrate that treatment with the herbs resulted in an increase of cells in the sub G_0_ phase of the cell cycle (Figure 5A) and an increase the pro-apoptotic caspase 3/7 activity (Figure 5B). Data summarized from [14,15,16].

Thus, modulations in the sub G_0_ population and caspase 3/7 activity by the herbs provide evidence for induction of cellular apoptosis in the present TNBC model. The data from the two end points provided a rank order of DA > CO > PC. Furthermore, these data also provide a rationale to examine the status of select pro-apoptotic or anti-apoptotic molecules that may function as potential molecular targets for the efficacy of the herbs.

The herbal formulas used for patients in the traditional Chinese medicine are prepared from a mixture of several herbs, and individual herbs may have multiple water soluble constituents. The clinical efficacy of the herbal formulas is predominantly due to synergistic interaction of potential bioactive constituents. The efficacy of herbs used in the present TNBC model is likely due to multiple water soluble bioactive agents present in the non-fractionated aqueous extract, and therefore cannot be ascribed to any specific bioactive agent.

## 3. Conclusions

The data for the present model system validates an experimental approach to evaluate Chinese herbs for their growth inhibitory efficacy against prevention of TNBC. Systemic toxicity and acquired tumor resistance represent commonly encountered limitations in chemotherapy and molecularly targeted therapy. These limitations suggest a lack of effective conventional therapeutic options for TNBC [49], and thereby emphasize the significance of identification of naturally-occurring nontoxic testable alternatives for secondary prevention/therapy of TNBC. In this context it is notable that sulforaphane, a bioactive agent present in broccoli and isothiocyanate present in cruciferous vegetables have documented inhibitory efficacy against breast cancer stem-like cells in vitro and in vivo [24,50]. Herbal formulations containing a mixture of several herbs are used in traditional Chinese medicine. These formulations affect complex biological activities that are relevant to cancer progression. Chinese herbs function as estrogenic, anti-inflammatory, anti-angiogenic and immune-modulatory agents, affecting multiple signaling pathways [51]. In TNBC these herbs are effective via inhibition of PI3K, AKT, mTOR, MAPK and Wnt/β-catenin signaling pathways [52]. Growth inhibitory effects of herbal saponins involve the PI3K/AKT/mTOR pathways in a TNBC model [53], and berbamine used in traditional Chinese medicine inhibits cellular proliferation, migration and invasion via the PI3K/AKT/MDM2/p53/mTOR pathways in a TNBC model [54]. Ginsenoside represents a potent bioactive agent present in the Panax ginseng herb. In the MDA-MB-231 TNBC model ginsenoside functions as an anti-proliferative/pro-apoptotic agent [55,56], as an anti-invasive agent [57,58], and as an anti-metastatic agent [59,60]. Most of the inhibitory effects of Ginsenoside involve PI3K, AKT, mTOR, EGFR-MAPK and STAT-3/NFkB signaling pathways. In addition, ginsenoside reverses paclitaxel resistance and augments doxorubicin induced apoptosis in TNBC models [61,62]. Thus, in addition to the growth inhibitory efficacy of the Chinese herbs discussed in the present review, published evidence suggests that in vitro and in vivo inhibitory effects of Chinese herbs involve multiple signal transduction pathways and multiple molecular targets.

The published evidence generated from the cellular models for the Luminal A subtype [11,12,13] and the TNBC subtype [11,12,13,14,15,16] provide mechanistic leads for the efficacy of Chinese herbs in breast cancer subtypes that differ in their status of hormone receptors. The growth inhibitory effects of Chinese herbs at relatively low concentrations suggest their potential significance as testable alternatives against prevention of therapy-resistant breast cancer. In this context, it needs to be recognized that in vivo translation of the in vitro data represents a speculative extrapolation. Evidence based translation will only be possible by future experiments involving transplantation of TNBC cells in to appropriate recipients and analysis of the tumors formed.

Future prospects: The present review provides a scientifically robust foundation for investigations designed to identify efficacious Chinese herbs that selectively inhibit growth of cancer cells.

Complex herbal formulas contain combinations of several herbs, each with multiple potential active agents. Thus, investigations focused on identification of relevant mechanisms and potential molecular targets for efficacy are faced with formidable scientific issues. Research directions focused on chemical composition of the herbs, network pharmacology, structure-activity studies and possible synergistic interactions between individual bioactive agents are likely to provide important mechanistic leads, and thereby, may overcome the limitations of scientific/technical issues.

Promising herbs could be prioritized for in vivo studies using the xeno-transplant model to evaluate the status of absorption, distribution, metabolism and excretion (ADME) and their pharmaco-kinetic patterns in the tumor-bearing recipients. These directions are likely to provide a basis for in vivo safety, efficacy and tumor selectivity of the herbs. Ex-vivo models from TNBC patient-derived tumor explants and tumor organoids [63,64] represent additional research directions that may provide a clinically translatable, mechanism-based rationale for future investigations focused on the applicability of Chinese herbs for secondary prevention/therapy of the TNBC patient.

## Figures and Tables

**Table 1:** Hyper-proliferation in the TNBC model.

Proliferation End Point	Cellular Model		*p*-Value	Relative to 184-B5
	184-B5	MDA-MB-231		
PDT (h) ^a^	34.1 ± 1.7	15.0 ± 2.2	0.040	−56.0%
Sat. Den. (×10^5^) ^b^	23.7 ± 1.3	32.9 ± 2.3	0.048	+38.8%
G_1_: S + G_2_/M ^c^	1.8 ± 0.3	0.6 ± 0.3	0.030	−66.7%
AI Colony Number ^d^	1.2 ± 1.0	750.0 ± 76.0	<0.001	+624×

^a^ determined from the exponential growth phase at day four after seeding of 1.0 × 10^5^ cells. ^b^ determined as viable cell number at day seven after seeding of 1.0 × 10^5^ cells. ^c^ determined from cell cycle analysis at day four after seeding. Data expressed as mean ± SD, *n* = 3 per treatment group and analyzed by Student *t* test. ^d^ determined at day 21 after seeding of 0.5 × 10^6^ cells by AI growth assay. Data expressed as mean ± SD, *n* = 3 per treatment group and analyzed by Student *t* test. PDT, population doubling time; Sat. Den., saturation density; AI, anchorage independent; SD, standard deviation. Data summarized from [14,15,16,17,18,19,20,21,22,23].

**Table 2:** Chinese herbs screened in the TNBC model.

Herb	Origin	Bio-Active Agent	Reference
Cornus officinalis (CO)	fruit	anthocyanins	[11,14]
Cuscuta sinensis (CS)	seed	flavonoids	[12,19], personal communication
Dipsacus asperoides (DA)	root	saponins	[12], personal communication
Drynaria firtunie (DF)	bark	flavonoids	[12,19,24], personal communication
Epimedium grandiflorum (EG)	Leaf, stem	icariin, icaritin, prenylflavone	[12,13]
Eucommia ulmoides (EU)	bark	lignans, tanins, saponins	[12,19]
Lycium barbarum (LB)	bark	lignans, tanins, saponins	[12,19], personal communication
Ligustrum lucidum (LL)	fruit	terpenoids	[12]
Psoralea corylifolia (PC)	seed	Coumarins, flavonoids, meroterpenes	[15]
Smilax glabra (SG)	bark	Falvonoids, saponins	[24], personal communication
Taraxacum mangolicum (TM)	leaf	flavonoids	[24], personal communocation
Viola yeodoensis (VY)	leaf	terpenoids	[24], personal communication

**Table 3:** Ranking of growth inhibitory efficacy of Chinese herbs by IC_50_.

Herb	Inhibitory Concentration (IC_50_ µg/mL) ^a^
CO	1.0 ± 0.3
PC	6.0 ± 1.1
DA	15.0 ± 3.7
LB	17.7 ± 4.5
VY	18.0 ± 4.5
EU	20.9 ± 5.2
LL	87.6 ± 21.7
CS	90.4 ± 22.4
EG	102.4 ± 25.6
DF	650.0 ± 24.4
TM	800.0 ± 20.7
SG	>1000

^a^ Non-fractionated aqueous extract. IC_50_ concentrations are extrapolated from the primary dose response in a seven-day growth assay. Data are presented as mean ± SD, *n* = 3 per treatment group.

**Table 4:** Ranking of growth inhibitory efficacy of Chinese herbs by IC_90_.

Herb	Inhibitory Concentration (IC_90_ µg/mL) ^a^
CO	5.0 ± 1.5
PC	20.0 ± 3.5
DA	30.0 ± 4.0
LB	32.0 ± 4.0
EU	38.0 ± 5.0
VY	200.0 ± 17.5
LL	274.0 ± 24.0
CS	671.0 ± 26.0
DF	1000.0 ± 13.6
EG, SG, TM	>1000

^a^ Non-fractionated aqueous extract. IC_90_ concentrations are extrapolated from the primary growth inhibitory dose response in a seven-day growth assay. Data are presented as mean ± SD, *n* = 3 per treatment group.

**Table 5:** Inhibition of anchorage independent colony formation.

Treatment	AI Colony Number ^a^	*p*-Value	% Inhibition
Control	750 ± 76		----
DA	55 ± 6	0.001	92.7
PC	69 ± 7	0.001	90.8
CO	120 ± 12	0.001	84.0
LL	339 ± 34	0.042	54.8
DF	372 ± 38	0.042	50.4
SG	373 ± 37	0.042	50.3
LB	373 ± 36	0.042	50.3
VY	375 ± 36	0.042	50.3
EU	396 ± 40	0.046	47.2
TM	396 ± 38	0.046	47.2
CS	649 ± 66	NS	13.5
EG	716 ± 73	NS	4.5

^a^ determined at day 21 after seeding on 0.5 × 10^6^ cells by AI growth assay. Data expressed as mean ± SD, *n* = 3 per treatment group and analyzed by one-way ANOVA with Dunnett’s multiple comparison test (α = 0.05). AI, anchorage independent; ANOVA, analysis of variance; NS, not significant.

## Data Availability

Data is contained within the article.
